# Combined Gamma Conglutin and Lupanine Treatment Exhibits In Vivo an Enhanced Antidiabetic Effect by Modulating the Liver Gene Expression Profile

**DOI:** 10.3390/ph16010117

**Published:** 2023-01-13

**Authors:** Paloma Lucía Guerra-Ávila, Tereso J. Guzmán, José Alfredo Domínguez-Rosales, Pedro Macedonio García-López, Alejandra Beatriz Cervantes-Garduño, Michael Wink, Carmen Magdalena Gurrola-Díaz

**Affiliations:** 1 Departamento de Biología Molecular y Genómica, Centro Universitario de Ciencias de la Salud, Instituto de Investigación en Enfermedades Crónico-Degenerativas, Instituto Transdisciplinar de Investigación e Innovación en Salud, Universidad de Guadalajara, Sierra Mojada 950, Col. Independencia, Guadalajara 44350, Jalisco, Mexico; 2 Department of Pharmacology, Institute of Pharmaceutical and Medicinal Chemistry, University of Münster, Corrensstraße 48, 48149 Münster, Germany; 3 Laboratorio de Productos Bióticos, Departamento de Botánica y Zoología, Centro Universitario de Ciencias Biológicas y Agropecuarias, Universidad de Guadalajara, Carretera Guadalajara-Nogales Km. 15.5, Las Agujas, Zapopan 44171, Jalisco, Mexico; 4 Laboratorio de Genómica Clínica, Facultad de Odontología, Universidad Nacional Autónoma de México, Coyoacán, Mexico City 04510, Jalisco, Mexico; 5 Institute of Pharmacy and Molecular Biotechnology, Heidelberg University, 69120 Heidelberg, Germany

**Keywords:** plant proteins, quinolizidine alkaloids, high-fat diet plus streptozotocin, type 2 rat diabetes (T2D), *Lupinus albus*, affymetrix microarray

## Abstract

Previous studies have individually shown the antidiabetic potential of gamma conglutin (Cγ) and lupanine from lupins. Until now, the influence of combining both compounds and the effective dose of the combination have not been assessed. Moreover, the resulting gene expression profile from this novel combination remains to be explored. Therefore, we aimed to evaluate different dose combinations of Cγ and lupanine by the oral glucose tolerance test (OGTT) to identify the higher antidiabetic effect on a T2D rat model. Later, we administered the selected dose combination during a week. Lastly, we evaluated biochemical parameters and liver gene expression profile using DNA microarrays and bioinformatic analysis. We found that the combination of 28 mg/kg BW Cγ + 20 mg/kg BW lupanine significantly reduced glycemia and lipid levels. Moreover, this treatment positively influenced the expression of *Pdk4*, *G6pc*, *Foxo1*, *Foxo3*, *Ppargc1a*, *Serpine1*, *Myc*, *Slc37a4*, *Irs2*, and *Igfbp1* genes. The biological processes associated with these genes are oxidative stress, apoptosis regulation, and glucose and fatty-acid homeostasis. For the first time, we report the beneficial in vivo effect of the combination of two functional lupin compounds. Nevertheless, further studies are needed to investigate the pharmacokinetics and pharmacodynamics of the Cγ + lupanine combined treatment.

## 1. Introduction

Type 2 diabetes (T2D) is a chronic metabolic disease that represents a growing public health challenge worldwide. Hyperglycemia, pancreatic β-cell dysfunction, insulin resistance, and dyslipidemia characterize T2D, the most frequent type of diabetes. In addition, obesity is an important risk factor in its development [1]. Currently, T2D management presents several approaches. Conventional T2D treatments involve dietary recommendations and treatment with biguanides, α-glucosidase inhibitors, glucagon-like peptide 1 receptor (GLP-1R) agonists, dipeptidyl peptidase 4 (DPP-4) inhibitors, sodium/glucose cotransporter 2 (SGLT2) inhibitors, insulin secretagogues (sulfonylureas or non-sulfonylureas), thiazolidinediones, and exogenous insulin. However, the permanent use of these drugs exhibits collateral effects, which may eventually deteriorate the functionality of several organs. Another relevant limitation consists of the high economic burden of novel drugs restricting the treatment adherence in many diabetic patients [2]. Therefore, the investigation, development, and production of new antidiabetic treatments, which may aid in the prevention and management of T2D, are important.

In recent years, the antidiabetic effect of natural compounds has gained scientific interest. In this regard, phytochemicals, bioactive compounds, and nutraceuticals exert promising properties for therapeutic application in the T2D treatment. In fact, the strategic inclusion of these natural compounds in the T2D management could be highly effective in prevention, diabetes control, and/or complementing the current T2D therapies [3]. Interestingly, lupins are a novel alternative that not only represent a legume with notable nutritional properties, but also contain bioactive compounds and nutraceuticals with beneficial health effects [4]. *Lupinus albus* (*L. albus*) seeds contain ~40% protein approximately distributed in albumins and globulins. Regarding globulins, four main fractions have been described: α, β, γ, and δ conglutins [5]. Cγ is a 7S basic protein fraction that represents about 5% of total globulins in *L. albus* seeds, and it is an oligomer composed of four or six monomers. Each monomer has a molecular weight of ~49 kDa and consists of two subunits (~30 kDa and ~18 kDa, respectively) linked by disulfide bridges [5,6,7,8].

Due to its antidiabetic properties reported in cellular models, animal experiments, and clinical studies, Cγ represents an important plant protein for human health improvement [5,9,10,11,12]. Furthermore, recent findings have indicated its influence on lipid metabolism, hypertension control, antioxidant activity, and autophagy [13,14,15,16,17,18,19,20,21]. In vitro and fluorescence polarization (FP) studies have suggested that Cγ interacts with insulin [9,22]. In addition, it stimulates the activation of the protein kinase C (PKC) generating a mimetic effect of insulin and increasing the translocation of the type 4 glucose transporter [20,23]. Likewise, this protein increases glucose uptake in HEPG2 cells and enhances the effects of insulin and metformin [24]. In in vivo assays, it was demonstrated that Cγ treatment does not produce hypoglycemia [10]. In pancreatic tissue, chronic treatment of Cγ in animals demonstrated an increase in insulin gene expression, insulin tissue content, and serum insulin levels [11]. Moreover, Cγ decreases in vivo the liver gene expression of glucose-6-phosphatase (G6PC), a key enzyme in gluconeogenesis [12]. These findings ratify the therapeutic potential of Cγ to control hyperglycemia, dyslipidemia, and oxidative stress.

On another hand, all lupin species contain different levels of quinolizidine alkaloids, which are toxic bitter substances detected in stems, leaves, and lupin seeds. Domesticated lupin species such as *L. luteus*, *L. angustifolius*, *L. mutabilis*, and *L. albus* contain very low alkaloid amounts. One of the major quinolizidine alkaloids contained in lupin species is lupanine [25,26,27,28,29]. In particular, several pharmacological activities have been reported for lupanine such as antimicrobial properties against Gram-positive and Gram-negative bacteria, and fungicidal properties against *Aspergillus niger* [30,31,32]. Furthermore, lupanine also has anti-inflammatory, antiarrhythmic, hypotensive, and hypoglycemic activities [33,34,35]. In experimental studies that evaluated the toxicity of lupanine in rats, a lethal dose 50 (LD_50_) of 1464 mg/kg BW by oral intake was reported [36]. Indeed, we must underline that, in the present study, the lupanine dose (20 mg/kg) was 80-fold lower than the reported LD_50_. This dose was able to decrease high glucose levels without causing hypoglycemia. Moreover, the antidiabetic activity exerted by lupanine was attributed to K_ATP_ channel blockade and insulin secretion [37]. Given this knowledge, lupanine has been proposed as a potential compound to be integrated into diabetes management, specifically due to its proven insulin secretagogue effect on pancreatic β cells only under hyperglycemic conditions.

Therefore, in this work, our interest was to evaluate the antidiabetic effect of a combined treatment, a protein fraction (Cγ) plus a quinolizidine alkaloid (lupanine), both present in lupins. Once we evaluated different Cγ plus lupanine doses in glucose response curves, we identified the best dose of the combination for further analysis. Next, we treated T2D-induced rats with the dose combination and assessed biochemical parameters, as well as the hepatic gene expression profile, and we compared these results against the data obtained from untreated T2D induced rats and from healthy animals.

## 2. Results

### 2.1. Characterization of Gamma Conglutin and Lupanine

The characterization of lupanine was performed by thin-layer chromatography (TLC). For the TLC, aluminum plates covered with silica gel 60 GF254 (Merck, Darmstadt, Germany) were used and revealed using the Dragendorff reagent. Likewise, lupanine was detected by TLC in the serum and urine of rats previously treated with this alkaloid (Figure S1). Additionally, the purity of lupanine was evaluated in GC–MS, and the spectra of lupanine are shown in Figure S2.

In accordance with our and other previous studies [11,12,21,38], we corroborated the isolation of gamma conglutin (Cγ). After SDS-PAGE characterization, we observed a band of 49 kDa, which corresponds to the protein in nonreducing conditions, and two subunits of ~30 and ~17 kDa under reducing conditions (Figure S3).

### 2.2. Acute Treatment Effects of Different Cγ and Lupanine Doses Evaluated by Oral Glucose Tolerance Test (OGTT)

Both lupin constituents, Cγ and lupanine, have been reported to exert antidiabetic activity; however, whether its combination produces an enhanced effect on blood glucose levels has not been reported. To investigate the influence of the combination of these compounds on glycemia levels, we performed the OGTT in healthy and experimentally T2D-induced rats treated acutely with different doses of the compounds mentioned above (Table S1).

#### 2.2.1. In Healthy Rats, an Enhanced Antidiabetic Effect Was Elicited by the Combination of Cγ (28 mg/kg BW) + Lupanine (20 mg/kg BW)

**Figure 1:**
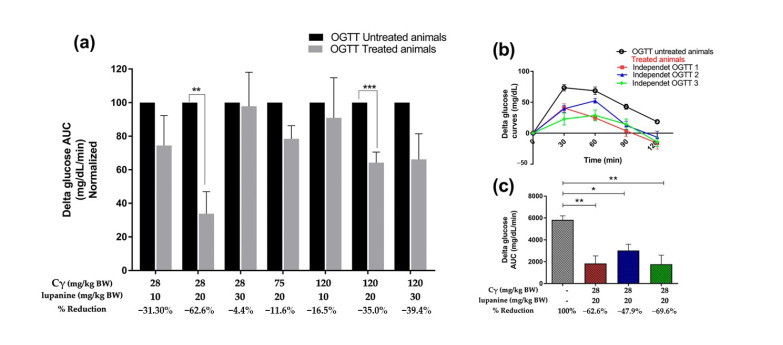
Glycemia reduction effect (percentage) of the different dose combinations of Cγ + lupanine. (**a**) OGTTs were performed with and without treatment in the same animal group. Delta glucose AUC values from untreated rats were normalized as 100%. Consequently, delta glucose AUC values from treated rats and the effect on glycemia reduction were calculated. (**b**) Glycemia reduction effect of 28 mg/kg BW Cγ + 20 mg/kg BW lupanine on healthy rats (*n* = 4) was confirmed in three independent experiments. Delta glucose curves obtained from OGTTs. (**c**) Delta glucose AUC (percentage) of three independent assays. Data were analyzed using a paired *t*-test. * *p* < 0.05, ** *p* < 0.01, *** *p* < 0.001.

For an objective comparison among the tested combination doses, we analyzed the AUC of the glycemia after the administration of an oral glucose load. The delta glucose AUC values of the control assays (without treatment) were used as a reference (100%) and compared to the delta glucose AUC of the treatment assays to evaluate the antidiabetic effect of each combinatorial treatment of Cγ + lupanine in different doses (Figure 1a). The effect of glycemia reduction (percentage) can be observed when the delta glucose AUC values of the treated groups were compared to those of the controls. To confirm the glycemia reduction effect observed in the previous screening, we performed three independent assays of the dose combination selected (28 mg/kg BW Cγ + 20 mg/kg BW lupanine) in healthy rats (*n* = 4). A reproducible effect was confirmed in these independent assays (average = −60.03%) (Figure 1b,c).

#### 2.2.2. In Diabetic Rats, the Glycemia Reduction Effect of the Treatment Combination of Cγ + Lupanine Was Similar to the Pharmacological Effect of a Conventional Treatment (Metformin + Glibenclamide)

**Figure 2:**
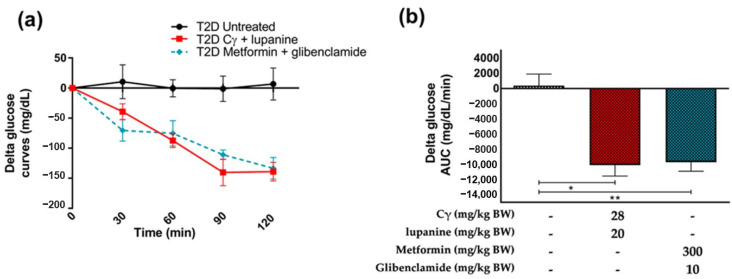
Comparison of acute treatment effects of Cγ + lupanine versus metformin + glibenclamide. (**a**) Follow-up of blood glucose concentration in response to the treatments: 28 mg/kg BW Cγ + 20 mg/kg BW lupanine and 300 mg/kg metformin + 10 mg/kg glibenclamide in diabetic rats, *n* = 4. Delta glucose curves at the different evaluated timepoints (**b**) Delta glucose AUC values. Data were analyzed using a paired *t*-test. * *p* < 0.05, ** *p* < 0.01.

In accordance with our previous results, we analyzed the combination of the 28 mg/kg BW Cγ + 20 mg/kg BW lupanine in T2D rats. Then, we decided to explore if this result was comparable with the antidiabetic effect elicited by a conventional drug combination (300 mg/kg BW metformin + 10 mg/kg BW glibenclamide). As expected, the conventional treatment importantly reduced glycemia. Interestingly, we observed a similar effect in the combination of Cγ and lupanine (Figure 2).

### 2.3. Chronic Treatment Effects

Once we selected a combination dose of our compounds, we decided to assess the influence of these lupin constituents on body weight, serum biochemical analytes, and the gene expression profile after a 7-day treatment period.

#### 2.3.1. Diabetic Rats Exhibited Body Weight Loss after 7 Days of Treatment (28 mg/kg BW Cγ + 20 mg/kg BW Lupanine)

**Figure 3:**
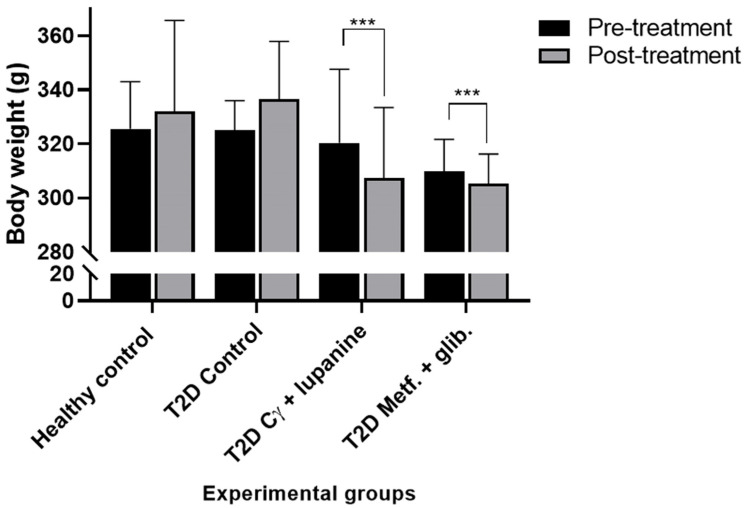
Comparison of body weight change among the experimental diabetic groups. In the untreated groups (healthy and diabetic animals), the body weight increased by 13 g and 11 g, respectively. In contrast, T2D rats that received the combination of our compounds showed a diminution of 13 g. Similarly, the rats that received the pharmacological treatment diminished their body weight by 4 g. Paired *t*-test between the body weight of animals on the first and last day of treatment, *n* = 10. *** *p* > 0.0001.

In Figure 3, we show body weight changes of rats induced by diabetes and treated with our compound combination. At the end of the treatment, the group that received the combination of 28 mg/kg BW Cγ + 20 mg/kg BW lupanine exhibited a body weight diminution in comparison with their basal body weight.

#### 2.3.2. Biochemical Parameters in Diabetic Rats Treated with Cγ + Lupanine

As expected, all T2D rat groups exhibited a notable blood glucose increase after the induction. With regard to lipid profile, we observed alterations in lipid analytes from T2D rats, due to the selected T2D model. T2D rats that received the combination of 28 mg/kg BW Cγ + 20 mg/kg BW lupanine showed a post-treatment decrease in triglycerides, as observed in T2D rats treated with the pharmacological combination of 300 mg/kg metformin + 10 mg/kg glibenclamide. Regarding the transaminase levels, AST and ALT, all T2D experimental groups showed an increase concentration indicating pathological changes in the liver tissue. Lastly, the urea and creatinine levels were analyzed to biochemically assess the renal function (Table 1).

### 2.4. Influence of the Cγ + Lupanine Combination on the Liver Gene Expression Profile of Diabetic Rats

After the 7-day treatment period, RNA isolation from the liver of diabetic rats was performed for further DNA microarray analysis. The gene expression profile in three experimental groups was explored: (1) healthy untreated animals, (2) T2D untreated animals, and (3) T2D animals treated with Cγ + lupanine combination. Firstly, the RNA integrity from every sample (before pools) was evaluated in order to confirm an optimal RNA quality and further proceed with the microarray analysis. All samples yielded an RNA integrity number (RIN) > 8.8 that allowed us to continue with the DNA microarray analysis (Figure S4). Gene expression data were analyzed using the specific Affymetrix software (Transcriptome Analysis Console, TAC, v.4.0.2, ThermoFisher Scientific, Santa Clara, CA, USA). In an initial exploration of condition pairings, the top 10 upregulated and downregulated genes were identified. Next, comparing all experimental groups, graphs of sample signals were generated, provided by TAC. We detected the strongly influenced genes by Cγ + lupanine treatment. Then, we performed a functional enrichment analysis in STRING database, and the results were focused on the identification of specific targets involved in carbohydrate and lipid metabolism.

#### Transcriptome Analysis Console v. 4.0.2

**Figure 4:**
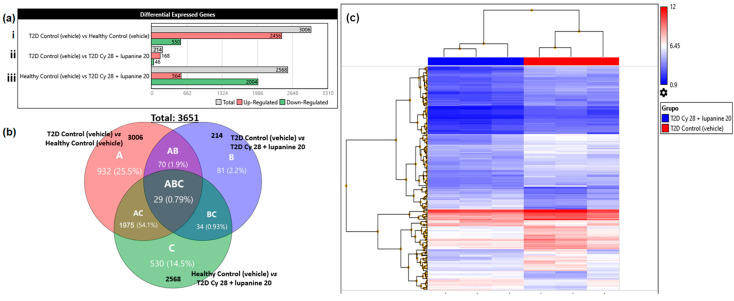
Overview of upregulated and downregulated genes in the experimental groups. (**a**) Differentially expressed genes comparing (**ai**) the T2D control vs. healthy control, (**aii**) T2D control vs. T2D Cγ + lupanine, and (**aiii**) healthy control vs. T2D Cγ + lupanine (**b**) Venn diagram depicting the number of DEGs among the experimental groups. (**c**) Hierarchical clustering analysis of comparison between T2D Cγ + lupanine group in blue color and the T2D untreated group in red color.

For the bioinformatics analysis, we used the transcriptome analysis console (TAC) and obtained from the gene expression files a total signal of 68,842 genes. When filter criteria were established (fold change >2 or <−2, *p* < 0.05), 30,932 differentially expressed genes (DEGs) were identified. Initially, we evaluated how the experimental diabetes conditions impacted the liver gene expression profile of healthy animals (Figure 4(ai)). Moreover, the influence of combined treatment on diabetic animals over healthy animals was also observed (Figure 4(aiii)). Nevertheless, we decided to focus on the analysis between T2D untreated and T2D treated with Cγ + lupanine animals (Figure 4(aii)). Regarding this comparison, the gene expression data remaining after filter criteria were as follows: 214 DEGs (214/68842, 0.31%), of which 168 (78.5%) genes were upregulated and 46 (21.5%) genes were downregulated (Figure 4(aii)). An additional filter of DEG was applied to include only up- and downregulated coding genes, and the results yielded 76 upregulated and 11 downregulated coding genes (data not illustrated).

On the other hand, the number of DEGs among the experimental groups is depicted in a Venn diagram (Figure 4b). A validation of the Cγ + lupanine effect in the reproducibility of every independent triplicate is shown in Figure 4c. The homogeneity of every triplicate was evaluated through the hierarchical clustering analysis. This analysis only allowed the comparison of two conditions (T2D Cγ + lupanine group in blue color and the T2D untreated group in red color) with three triplicates (considering every triplicate as a pool). Therefore, it is observed that the T2D model, as well as the treatment, strongly maintains uniformity and is mostly reproducible.

Next, we identified the upregulated and downregulated coding genes that presented the greatest fold change values. In Table 2, we include which genes were more regulated (up- and down-) after treatment with Cγ + lupanine.

In order to know which genes reestablished their expression levels under the influence of Cγ + lupanine treatment, we compared the individual sample signals (expression level) from each gene generated in the transcriptome analysis console (TAC). For this analysis, we included only coding genes (12,551). The graph of sample signals shows the expression levels of an individual gene in each group (Figure S5). The graphs corresponding to each selected gene were compared in all animal groups: healthy control group, T2D control group, and T2D with the combination Cγ + lupanine group (Table 3).

Lastly, a functional enrichment analysis was generated in STRING to evaluate the relationship between targets identified in Table 3. Later, an analysis of functional enrichments was performed in the network (59 targets), provided by Gene Ontology (GO), KEGG pathways, and WikiPathways. According to GO analysis, the main related biological processes were 57 from 59 total targets in cellular processes, 44 response to stimulus, 37 response to chemical, 36 multicellular organismal processes, 33 response to organic substance, 31 anatomical structure development, 27 regulation of molecular function, 26 regulation of biological quality, 23 regulation of signaling, 22 regulation of signal transduction, 23 regulation of cell communication, 20 regulation of apoptotic processes, and 18 homeostatic process.

**Figure 5:**
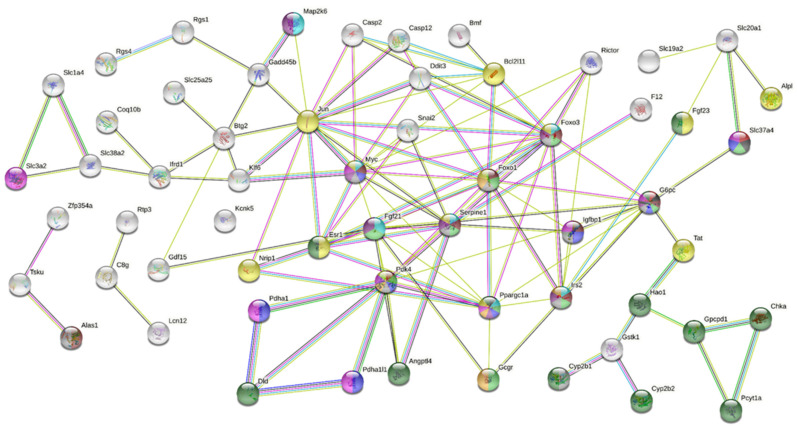
Functional enrichment analysis by STRING. Biological processes, color (pantone hex code): gluconeogenesis, dark gray (#53565a); glucose-6-phosphate transport, red (#c1232d); glucose metabolic process, blue (#0026ab); glucose homeostasis, light green (#9dd38b); response to carbohydrate, purple (#634177); cellular response to carbohydrate stimulus, turquoise (PMS 3115 C; #00c1d4); regulation of cellular carbohydrate metabolic process, yellow mustard (#e1ad01); carbohydrate metabolic process, pink (#d62598); cellular response to insulin stimulus, ruby wine (#77353d); response to insulin, light gray (#c5c6c7); response to lipid, yellow (#ffe957); lipid metabolic process, dark green (#284d1c).

Afterward, we decided to focus on the analysis of targets involved in carbohydrate and lipid metabolism. These targets are highlighted in different colors (according to the biological process) in Figure 5. Interestingly, with regard to the molecular function identified with the GO analysis, we found that three of the targets were related to the pyruvate dehydrogenase activity. In the same analysis, we screened which cellular components are related to our identified targets. Overall, we found that 48 of 59 total targets were presented in the cytoplasm: 16 in the mitochondrion, seven in the mitochondrion matrix, and three in nuclear euchromatin.

On the other hand, we explored the cellular pathways related to our targets. Using the Kyoto Encyclopedia of Genes and Genomes (KEGG), we performed an analysis of the pathways and detected: 12 targets involved in metabolic pathways, eight in cancer, seven in MAPK signaling, six in PI3K/Akt signaling, six in Foxo, six in apoptosis, five in AMPK signaling, five in glucagon signaling, and four in insulin resistance.

According to the WikiPathways database, eight targets were involved in adipogenesis, along with six in MAPK signaling, five in insulin signaling, four in nuclear factor erythroid-derived 2-like 2 (Nrf-2) signaling, four in IL-6 signaling, four in apoptosis, four in hexose metabolism in proximal tubules, three in p38 MAPK signaling, three in tricarboxylic acid (TCA) cycle, and two in insulin-induced PI3K/Akt and MAPK in hepatocytes.

## 3. Discussion

Lupins are legumes that have captured attention over the past years for their nutritional properties, traditionally regarding a higher protein content (up to ~40%). It is widely accepted that lupin bioactive compounds and nutraceuticals can exert novel and beneficial effects on animal and human health [5,39,40].

In 2004, it was reported, for the first time, a significant reduction in blood glucose levels after Cγ oral administration (30, 60, and 120 mg/kg BW) in rats, and the effect was dose-dependent. In addition, a similar effect was observed on glucose reduction in animals treated either with Cγ (120 mg/kg BW) or with metformin (50 mg/kg BW) [9]. On the other hand, the antidiabetic effect with other doses (28 mg/kg and 42 mg/kg BW) was also evaluated and confirmed [10,24]. Among the globulin protein fractions, the most studied has been Cγ; however, the biological activity of other conglutins such as beta conglutin (Cβ) remains to be further investigated. On the other hand, the different methodological aspects of the interventional studies with lupin whole seeds, protein isolates, or lupin fiber must be thoroughly analyzed in order to weigh their divergences in the biological effect [41,42].

Lupanine, another lupin bio-compound belonging to the group of quinolizidine alkaloids, exerts an antidiabetic effect, as demonstrated through in vitro and in vivo studies. Among these alkaloids, Garcia-Lopez and collaborators evaluated in mouse pancreatic islets the effects of lupanine, 13-α-OH lupanine, 17-oxo-lupanine, and 2-thionosparteine on insulin release at different glucose concentrations (3.3, 8.3, and 16.7 mmol). Interestingly, the results showed that 0.5 mM lupanine increased insulin secretion but only at high glucose concentrations, representing an advantage over sulfonylureas [43]. In 2015, Wiedemann and collaborators investigated in healthy and diabetic rats, the effect of the oral administration of 20 mg/kg BW lupanine (80 times lower than the reported LD50) [36]. After treatment, hypoglycemia was not observed either in healthy rats or in diabetic rats [37]. Therefore, in the first stage of our study, we selected different Cγ and lupanine doses and evaluated them independently, as well as in combination. Taken together, our results confirmed the antidiabetic effect of the individual treatments and were in agreement with the studies described previously. These data are also in line with the literature, where hypoglycemia was also not observed in animals after lupanine, Cγ or combined treatment (Table S1).

Previous studies have reported that the antidiabetic effect produced by lupanine lies in the blockade of ATP-dependent potassium channels, which is a crucial step for insulin release in β cells [37,43,44]. On the other hand, it has been shown that Cγ decreases the expression of glucose-6-phosphatase (*G6pc*), an enzyme responsible for the control of hepatic glucose production [12]. Regarding conventional diabetes treatment, drugs such as metformin and glibenclamide have been recommended as a common combination to manage T2D. Metformin constitutes first-line T2D treatment, and it has been demonstrated to decrease gluconeogenesis [45]. On the other hand, glibenclamide, a classical sulfonylurea, triggers insulin secretion by binding to the SUR1 subunit of the ATP-sensitive potassium (K_ATP_) channels, resulting in their closure in pancreatic β-cells [46].

After analyzing the effect of different doses combinations on glucose metabolism by OGTT, we identified the most effective combination (28 mg/kg BW Cγ plus 20 mg/kg BW lupanine). From our results, we speculated that both compounds in this combination effectively exert their antidiabetic effect on specific target organs (pancreas in the case of lupanine, and liver in the case of Cγ). It is worth to mentioning that this effect is even more visible in diabetic rats, since the effect of lupanine is displayed under high glucose concentrations. Regarding the chronic treatment effects with the selected dose, we focused on characterizing the biochemical parameters, as well as the liver gene expression profile among the evaluated groups.

The body weight of animals treated either with the combination of Cγ + lupanine or metformin + glibenclamide was significantly reduced after treatment. Our results are in agreement with previous studies that demonstrated a similar effect with individual treatments of glibenclamide or metformin in T2D-induced animals [47,48]. On the other hand, it has been also reported that lupin food products promote a reduction in body weight in animals and humans [49]. Interestingly, in our study, we tested Cγ, an isolated protein fraction, which possibly contributes to this effect. Whether lupanine also influences the body weight needs to be addressed in future studies.

Concerning biochemical parameters, one aspect that should be considered when comparing glucose values between different groups of experimentally induced animals is that their pretreatment values may vary among study groups. Among the reasons that can explain these differences, the biological variability of living organisms in response to experimental induction should be considered. In the same way, the random assignment of animals to the different experimental groups may also increase the variability. In our study, however, all the induced groups exhibited adequate glucose values for experimental diabetes in rats considering the established cutoff value (>200 mg/dL) [50]. Therefore, intragroup comparisons were performed between the pre- and post-treatment values of glucose, as well as the other biochemical analytes. In induced and treated animal groups with both combinations (Cγ + lupanine or metformin + glibenclamide), the animals displayed a reducing effect on glucose levels, in accordance with previous reports in the literature [12,37,51,52]. Compared to the conventional treatment with antidiabetic drugs, we found a decrease in triglyceride, cholesterol, and LDL-c levels after treatment with the combination of Cγ + lupanine. Other authors also reported a reduction in the parameters of the lipid profile, after treatment with lupin proteins or metformin, in similar experimental models [53,54]. In contrast, in the hepatic profile, we observed an increase in ALT and AST levels, explained by the liver damage caused by the induction model, as reported previously [55]. Lastly, treated animals with Cγ + lupanine showed an improvement in urea and creatinine, biochemical parameters related to renal function. In agreement with these results, an improvement of urea and creatinine levels was reported after treatment with the rutaecarpine alkaloid in a comparable experimental model (HFD + STZ) [56].

Since, in the diabetes pathophysiology, the liver represents a key organ in metabolism regulation, we decided to evaluate the hepatic gene expression profile. According to the bioinformatic analysis of our microarray results, we firstly identified different signaling pathways between both untreated groups (healthy and T2D animals), influenced only by the T2D experimental induction. Several studies have reported that oxidative stress, mitochondrial dysfunction, endoplasmic reticulum stress, inflammation, and lipotoxicity are relevant processes in the T2D development [57]. Remarkably, we identified genes in T2D-induced rats that restored partially their gene expression levels after the treatment with Cγ + lupanine. Such genes were related to different pathways: in the oxidative stress pathway, *Ddit3*, *Foxo1*, *Foxo3*, *Klf6*, *Ppargc1a*, and *Serpine1*; in the IL-6 signaling pathway, *Map2k6*, *Foxo1*, *Foxo3*, and *Jun*; in the Nuclear factor erythroid 2-related factor 2 (Nrf2) signaling pathway, *Map2k6*, *Slc1a4*, *Gstk1*, and *Jun*. These findings are in accordance with previous studies that analyzed the individual Cγ effect to reduce the proinflammatory response and insulin resistance in PANC-1 cells, through the modulation of INF-γ expression, cytokines (IL-6, IL-12P70, IL-17A, and IL-27), and ROS levels, as well as an increase in antioxidant molecules [20,58]. Furthermore, animal studies have reported that the intake of soybean proteins directly improves oxidative stress [59,60,61]. Even if studies addressing the antioxidant or anti-inflammatory effect of lupanine have not been conducted, some reports have evaluated the effects of different alkaloids showing a gene expression reduction in proinflammatory genes such as TNF-α, IL-6, and IL-1 followed by an increase in antioxidant enzymes due to Nrf2 signaling activation and a decrease in the profibrogenic transcription factor Snail-1 [62,63]. Therefore, we hypothesize that the Cγ + lupanine combination exerts synergized anti-inflammatory and antioxidant effects that allow the restoration of the expression of genes involved in the abovementioned processes.

In order to explain the observed metabolic and biochemical effects of animals treated with Cγ + lupanine treatment, we focused on identifying genes involved in the metabolism of lipids and carbohydrates which restored their gene expression levels to a healthy condition. We found that *Pdk4*, *G6pc*, *Foxo1*, *Foxo3*, *Ppargc1a*, *Serpine1*, *Myc*, *Slc37a4*, *Irs2*, and *Igfbp1* were relevant genes in these processes, partially reestablishing their expression levels after treatment. Our results are in accordance with previous studies with similar T2D induction models, which reported comparable findings in such genes involved in lipid and carbohydrate metabolism. It has also reported that one of the main regulators of gluconeogenesis is the transcriptional factor *Foxo1*, which binds to insulin-sensitive elements in the promoter of *Pdk4*, *G6pc*, and *Pepck* genes [64]. Moreover, it was reported that the response to insulin in the liver was related to the increase in *Foxo1* and *Foxo3* phosphorylation associated with the upregulation of *Gck* and the downregulation of *G6pc* and other canonical targets of *Foxo* in the liver, such as *Pck1*, *Igfbp1*, and *Ppargc1a* [65]. On the other hand, it has been reported that the modified gene expression of *Slc37a4* (6-phosphate glucose transporter, G6P) is related to the *de novo* stimulation of lipogenesis and the development of liver steatosis [66]. Regarding *Serpine1*, it was observed that, together with *Cdkn1a* and *Rprm*, it participates in the p53 signaling pathway and is involved in hepatocyte apoptosis during NAFLD development [67]. As another restored gene related to apoptosis, *Myc* is a regulator of cell proliferation, and the modification of its gene expression levels was related to T2D [68]. Moreover, some studies have shown a reduction in *Irs2*, a molecule involved in the insulin signaling after T2D induction [69,70,71]. Altogether, the restoration of the expression of these abovementioned genes by Cγ + lupanine treatment indicates an improvement of liver function, contributing to suppress the oxidative stress, endoplasmic reticulum stress, and apoptosis. These effects may also promote cell proliferation, normalization of autophagy, and regulation of mechanisms implicated in lipid and carbohydrate metabolism, which are altered in T2D. However, future studies might validate our current results by applying techniques such RT-qPCR and/or Western blot for further analysis of specific targets.

## 4. Materials and Methods

### 4.1. Plant Material

*Lupinus albus* certified seeds were kindly provided by E. van Santen (College of Agriculture, Auburn University, Auburn, AL, USA). Lupin seeds were dehulled and ground to flour. Next, lupin flour was hexane-defatted by reflux (36 h) using a Soxhlet equipment, according to Wink’s specifications [72,73].

#### 4.1.1. Extraction and Characterization of Bioactive Compounds from Defatted Lupinus Albus Flour

##### Isolation of the Quinolizidine Alkaloid, Lupanine

Briefly, the defatted lupin flour was dried for 24 h in a laboratory fume hood at room temperature; afterward, a 25% (KHO) solution was added to 300 g of this flour until a paste was obtained and allowed to stand for 3 h. Next, 300 g of diatomaceous earth (DE) was added, and the homogenized mixture underwent reflux with anhydrous ether for 14 h. The solvent was recovered using a rotary evaporator, and the residue with a high content of lupanine was used for obtaining lupanine perchlorate as follows: the residue was resuspended with 50 mL of methanol, and the pH was adjusted to 6.5 with perchloric acid and by the friction of the vessel walls with a glass rod the crystallization once initiated. It was allowed to stand for 12 h to evaporate the solvent. The ethanol solution containing the crystals leaked and was immediately washed with anhydrous ether.

##### Characterization and Detection of Alkaloids by Thin-Layer Chromatography (TLC)

Lupanine perchlorate crystals were resuspended in 1 mL of distilled water, and four drops of 1 M ammonium hydroxide and 1 mL of dichloromethane were added. The mixture was stirred and allowed to rest for a few minutes until two immiscible phases were distinguished; the lower phase (dichloromethane) was separated using a Pasteur pipette. Confirmation of lupanine isolation in the form of perchlorate was performed by thin-layer chromatography (TLC). For the TLC, aluminum plates covered with silica gel 60 GF254 (Merck) were used, with a mixture of dichloromethane, methanol, and NH_4_OH in a proportion of 8:2:0.5, while Dragendorff reagent was used to reveal the results [74].

##### Gamma Conglutin (Cγ) Extraction

The Cγ protein was extracted following a method described previously [11,12,21,38]. First, double-distilled water (ddH_2_O) was added to 20 g of defatted lupin flour (at a 1:10 flour-to-ddH_2_O ratio) to separate the albumin fraction. The suspension was stirred for 2 h at 4 °C. Next, it was centrifuged at 8000 rpm for 30 min at 4 °C, and the supernatant was eliminated. Then, the sediment was resuspended in a 10% NaCl (pH 7.0) and stirred for 12 h to 4 °C. The suspension was centrifuged in the same conditions, and the supernatant was recovered (globulin fraction). Next, the globulins were precipitated out by saturation with 85% ammonium sulfate and then centrifuged under the conditions described above. The pellet was dissolved in 0.1 M phosphate buffer (pH 6.8) and dialyzed in 0.2 M acetate buffer (pH 4.8) for 18 h. After this period, the mixture was centrifuged (8000 rpm for 30 min at 4 °C) to separate α conglutin contained in the sediment. The supernatant was dialyzed against ddH_2_O for 48 h at 4 °C. Finally, the solution was centrifuged (8000 rpm for 30 min at 4 °C), and the supernatant containing Cγ was lyophilized (Freeze Drying 4.5, LABCONCO) at −50 °C, 0.040 mbar, for 8 h.

##### Characterization of Cγ by SDS-PAGE

The Cγ isolate was characterized by 12% SDS polyacrylamide gel electrophoresis (SDS-Page). Protein samples (2 μg) were mixed with Laemmli sample buffer (Bio-Rad, Milan, Italy) with and without 1% β-mercaptoethanol (reducing and nonreducing conditions, respectively). They were incubated at 90 °C for 2 min and then centrifuged at 1000 rpm for 10 min at 4 °C. Electrophoresis in polyacrylamide was performed using the Protean^®^ Tetra Cell Mini-gel kit (Bio-Rad, Milan, Italy). The gels were stained with Coomassie brilliant blue G-250 (Bio-Rad, Milan, Italy). A Benchmark ™ Pre-stained protein ladder (Invitrogen, Loughborough, UK) was included to compare the relative molecular masses of native and reduced Cγ.

### 4.2. Preparation of Protein in Solution with Alkaloid

During the quality control when carrying out the OGTT assays in healthy rats, the following points were considered:

(a) Exact calculation of the amount of Cγ and lupanine needed for each study animal according to body weight.

(b) Cγ + lupanine solutions were prepared at different doses and dissolved in physiological saline solution (vehicle). Since the lupanine dose was very small, a lupanine stock solution (with a higher concentration) was prepared, and the corresponding alkaloid volume was calculated according to the body weight of each animal. Lupanine and Cγ volumes were mixed together for oral co-administration.

### 4.3. Animals

Male Wistar rats between 6 and 8 weeks of age (150–220 g body weight, BW) were obtained from the Universidad de Guadalajara bioterium. Animals were allowed to adapt for a period of 7 days, and then maintained at 24 ± 2 °C, 55% ± 5% humidity, and light/dark cycles of 12 h each. Water and standard rat diet were provided ad libitum. Research, Bioethics, and Biosecurity Committees of the Universidad de Guadalajara approved this study (CI-01919). All procedures were conducted in accordance with the International Guidelines for Care and Use of Laboratory Animals and the Mexican Official Standard 062 (update NOM-062-ZOO-2001).

### 4.4. Study Design

In this study, we assessed the acute and chronic effects of dose combinations of Cγ and lupanine. In a first stage, we performed the oral glucose tolerance test (OGTT) in heathy animals treated with a single dose combination of the compounds. With the results, we were able to identify the best dose combination. Then, we corroborated the observed acute effects of the selected dose combination in T2D animals.

**Figure 6:**
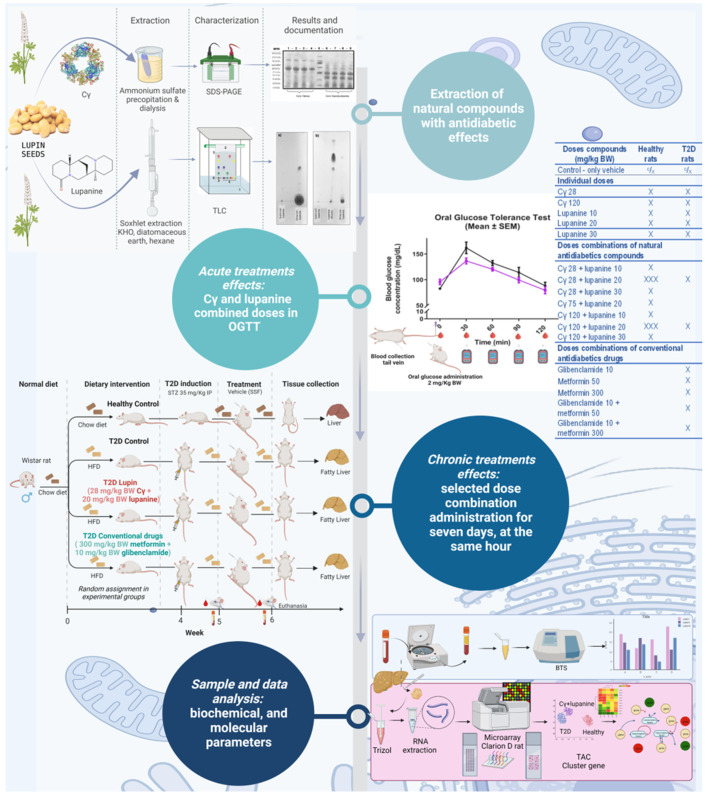
Study design.

Afterward, we administered daily the selected dose combination for a week in T2D animals. After this time period, we explored the gene expression profile using DNA microarrays and bioinformatic analysis of the results (Figure 6).

#### 4.4.1. Oral Glucose Tolerance Tests to Screen Cγ and Lupanine Combined Doses and Its Acute Treatment Effects

Our first aim was the identification of the best dose combination of Cγ and lupanine on glycemia in healthy and diabetic animals. Therefore, we administered a single dose of each compound and evaluated its antidiabetic effect using oral glucose tolerance tests (OGTTs). We decided to test the effect under physiologic and diabetic conditions (healthy and diabetic rats). During this acute treatment phase, we tested different doses of the compounds to identify the best dose combination that leads to a greater decrease in blood glucose levels. Male rats were randomly assigned to different groups (*n* = 4). Next, we performed the OGTT with and without treatment on each experimental group, meaning that each group was its own control during the experiments. In the control OGTT, the animals received only vehicle (0.9% *w*/*v* NaCl). In the OGTT with treatment, first, the compounds extracted from *Lupinus albus* were evaluated individually according to the minimum and maximum doses reported in the scientific literature: 28 mg/kg BW [24] and 120 mg/kg BW [9,11,38] Cγ, and 10, 20 [37], and 30 mg/kg BW lupanine, in healthy and diabetic rats. In addition, we evaluated the conventional pharmacological treatments for T2D, 300 mg/kg BW of metformin [12,75] and 10 mg/kg BW of glibenclamide [11], individually and combined in diabetic rats.

Several OGTT were performed with eight different combinations:

(1) 28 mg/kg BW Cγ + 10 mg/kg BW lupanine,

(2) 28 mg/kg BW Cγ + 20 mg/kg BW lupanine,

(3) 28 mg/kg BW Cγ + 30 mg/kg BW lupanine,

(4) 75 mg/kg BW Cγ + 20 mg/kg BW lupanine,

(5) 120 mg/kg BW Cγ + 10 mg/kg BW lupanine,

(6) 120 mg/kg BW Cγ + 20 mg/kg BW lupanine,

(7) 120 mg/kg BW Cγ + 30 mg/kg BW lupanine,

(8) 300 mg/kg BW metformin + 10 mg/kg BW glibenclamide.

Later, the results were analyzed and compared. We selected the dose combination comparable to the antidiabetic effect exerted by the metformin and glibenclamide combination. The treatment effects triggered by the different combination doses were selected according to their statistical significance in the area under the curve (AUC) analysis. This analysis including a comparison (when compared with the control without treatment), was performed in triplicate in healthy rats to confirm reproducibility. Finally, we also performed the OGTT in diabetic rats employing the best combinations to identify the most efficient dose combination. According to our results, the dose combination selected for the chronic phase was 28 mg/kg Cγ + 20 mg/kg lupanine.

##### Oral Glucose Tolerance Test (OGTT)

The OGTT was performed to identify the magnitude of the antidiabetic effect and the optimal doses, in both healthy and T2D rat groups. The animals were subjected to a fasting period of 12 h. Animals received the treatment or vehicle (0.9% *w*/*v* NaCl), 30 min before oral administration of a glucose solution (2 g/kg BW). The blood samples were collected from the tail vein, and blood glucose was measured with a standard glucometer (Accuchek, Roche, Germany) at 0, 30, 60, 90, and 120 min.

In the case of diabetic animals, we performed a similar protocol without the administration of the glucose load, to maintain the glycemia levels within glucose values detectable by the glucometer during the assay (<599 mg/dL).

##### Delta Glucose Curves

The data were normalized (calculating delta glucose values) by setting the baseline glucose values at zero and calculating the difference between each time interval. Due to the variations in baseline blood glucose, the delta glucose area under curve was calculated [76].

##### Delta Glucose AUC Values

The area under the curve (AUC) of each experimental group was calculated using the trapezoidal rule between 0 at 120 min, which represents the magnitude of the glucose response. The glucose response of treated animals was compared with their AUC when they received only vehicle (control, intra-treatment analysis). Then, the delta glucose AUC of each experimental condition was calculated as a percentage.

#### 4.4.2. Chronic Treatment Effects

To evaluate the chronic effect of the selected dose combination of Cγ and lupanine, we performed a 1 week treatment in different animal groups, and then evaluated biochemical and molecular parameters.

Animals were randomly assigned to four experimental groups (*n* = 10):

Healthy control group: rats receiving only vehicle (0.9% *w*/*v* NaCl),

T2D control group: diabetic rats with vehicle administration,

T2D lupin group: diabetic rats treated with the combination of 28 mg/kg BW Cγ + 20 mg/kg BW lupanine (both isolated from *Lupinus albus*), dissolved in the same vehicle,

T2D pharmacological group: diabetic rats treated with the combination of 300 mg/kg BW metformin + 10 mg/kg BW glibenclamide group dissolved in the same vehicle.

All animals were daily administered by oral gavage for seven consecutive days at the same hour.

### 4.5. T2D Model

The animals were induced to T2D by feeding a HFD including 60% fat of animal origin [50,77] for 4 weeks. Then, they were fasted for 12 h, received a single intraperitoneal (i.p.) injection of a low dose of STZ (35 mg/kg BW, diluted in a 0.1 M sodium citrate buffer, pH 4.5), and continued receiving HFD weekly [78,79,80]. At the fifth week, fasting glucose concentration was monitored using a glucometer (Accuchek, Roche, Germany). Animals exhibiting glycemia ≥200 mg/dL were considered as diabetic.

### 4.6. Blood Collection

The animals were fasted for 12 h and anesthetized with isoflurane. Peripheral blood samples were collected from the retro-orbital plexus. Serum was separated by centrifugation of blood samples (3500 rpm, 15 min, 4 °C) and stored in aliquots at −70 °C until analysis.

### 4.7. Biochemical Parameters

Serum concentration of biochemical analytes was determined by colorimetric assays using a semi-automated spectrophotometer (BTS-350; BioSystems, Barcelona, Spain). Serum levels of glucose, alanine aminotransferase (ALT), aspartate aminotransferase (AST), urea, creatinine, total cholesterol (TC), high-density lipoprotein cholesterol (HDL-c), low-density lipoprotein cholesterol (LDL-c), and triglycerides were quantified before (pre-) and after (post-) chronic treatment effects. All procedures were performed according to the manufacturer’s instructions (BioSystems, Barcelona, Spain).

### 4.8. RNA Extraction

The extraction of total liver tissue RNA was carried out using the RNeasy^®^ Mini Kit (Qiagen, Hilden, Germany), according to the manufacturer’s recommendations. The concentration and purity of the RNA were quantified by spectrophotometry using a Nanodrop 2000 spectrophotometer (ThermoFisher Scientific, Santa Clara, CA, USA). The integrity of the RNA was evaluated by microfluidic analysis using the Agilent 2100 Bioanalyzer (Agilent Technologies, Santa Clara, CA, USA) with an RNA LabChip^TM^ Kit (Agilent Technologies, Santa Clara, CA, USA) and denaturing agarose gel electrophoresis.

### 4.9. DNA Microarray

Three biological replicates of liver total RNA pools were prepared for each experimental group: healthy rat group, type 2 diabetic control group, and type 2 diabetic rats treated with 28 mg/kg BW Cγ + 20 mg/kg BW lupanine. Each pool consisted of the mixture of RNA isolated from three different animals.

A total of three technical replicates (derived from the RNA of nine animals per group) were processed for hybridization into the Affymetrix’s Clariom^TM^ D Rat (Affymetrix, Santa Clara, CA, USA) at the Microarray Unit of the National Institute of Genomic Medicine (INMEGEN), Mexico. The procedures of hybridization and scanning of the microarray were performed according to GeneChip^TM^ Whole-Transcript (WT) Expression Arrays User Guide of Affymetrix Corporation. Briefly, Clariom D rat is based on a photolithographic microarray platform with oligonucleotide sequences printed onto a glass substrate formatted cartridge. Initially, the synthesized first-strand cDNA was performed by reverse transcription, and total RNA was primed with primers containing a T7 promoter sequence at the 5′ end. The second-strand cDNA was synthesized by simultaneously using DNA polymerase and RNAase H to degrade the RNA. The second-strand cDNA was converted to double-stranded cDNA, which acted as a template to synthesized cRNA in vitro transcription (RT-IVT method). Samples were hybridized for 16 h at 4 °C. Then, cRNA purification and quantification were carried out, and enzymes, salts, inorganic phosphates, and unincorporated nucleotides were removed to prepare the cRNA for second cycle single-stranded cDNA (ss-cDNA) synthesis by reverse transcription of cRNA. RNase H was added to remove template RNA. The ss-cDNA was purified and fragmentation using uracil-DNA glycosylase (UDG) and apurinic/apyrimidinic endonuclease 1 (APE1) at the unnatural dUTP residues. The fragmented cDNA was labeled with terminal deoxynucleotidyl transferase (TdT) and biotinylated labeling reagents. Next, 5.2 µg of fragmented and biotinylated ss-cDNA was added to 160 µL of Hybridization Master Mix. Subsequently, the hybridization cocktails were prepared and heated at 99 °C for 5 min and 45 °C before injecting 200 µL into the Clariom D Rat array. The arrays were incubated in the GeneChip Hybridization Oven 645 and rotated at 60 rpm for 16 h at 45 °C. Immediately after hybridization, the arrays were stained and washed using the GeneChip Hybridization, wash, and stain kit (Thermo Fisher Scientific REF 900720) and GeneChip Fluidics Station 450. The array was scanned using a GeneChip Scanner 3000 7G System (Affymetrix, CA, USA). Array scanning was performed according to the manufacturer’s instructions (Affymetrix). Data files of type CEL and CHP were generated and processed with the Affymetrix software.

### 4.10. Bioinformatic Analysis

The gene expression files were processed and analyzed with the Applied Biosystems^TM^ Transcriptome Analysis Console 4.0.2 (TAC, ThermoFisher Scientific, Santa Clara, CA, USA). Gene expression data were RMA normalized, and differential expression was determined with the following parameters: RMA + DABG (*Rattus norvegicus*) and expression analysis according to fold change <−2 or >2, *p* < 0.05; ANOVA method ebayes. For the transcriptome analysis, group sample signals of each gene were compared to all groups to identify which genes were reestablished to a healthy gene expression level (exhibited by the healthy untreated group) after the Cγ + lupanine treatment. For functional enrichment analysis, STRING software was used to analyze differentially expressed targets interactions. Gene Ontology (GO), KEGG pathways, and WikiPathways analyses were performed to identify the main biological functions, cellular compartments, and pathways, as well as molecular networks, influenced by the treatment.

### 4.11. Statistical Analysis

The data were expressed as the mean ± standard error of the mean (SEM). Results from the area under the curve (AUC) of OGTT, and the intragroup differences (pre vs. post treatment) of the biochemical analytes were evaluated with the paired *t*-test. A *p*-value < 0.05 was considered significant. Graphing and calculation of AUC analysis were performed using GraphPad Prism Software version 8.0.1 (GraphPad Software, San Diego, CA, USA). Gene expression level data were analyzed according to the Affymetrix software.

## 5. Conclusions

Our findings showed that the combination of two lupin natural compounds, Cγ + lupanine, provides a promising beneficial effect for a future therapeutic application in the prevention or management of T2D. The dose combination with the highest antidiabetic effect was 28 mg/kg BW Cγ plus 20 mg/kg BW lupanine. Interestingly, our findings revealed that this combination significantly improved the hyperglycemia and lipid profile. The profile of gene expression levels shows that *Pdk4*, *G6pc*, *Foxo1*, *Foxo3*, *Ppargc1a*, *Serpine1*, *Myc*, *Slc37a4*, *Irs2*, and *Igfbp1* genes were restored, at different magnitudes, whereby their expression tended toward levels observed in a healthy condition. The main biological processes associated with these genes are oxidative stress, apoptosis regulation, and glucose and fatty-acid homeostasis. Additional studies will be helpful to corroborate our current results and contribute to a further understanding of the mechanism of action of this combined treatment. Research studies addressing pharmacokinetics and pharmacodynamics of Cγ and lupanine are also required, to establish their potential therapeutic application in humans.

## Figures and Tables

**Table 1:** Biochemical parameters pre- and post-treatment in different groups of rats.

	Experimental Groups							
	Healthy Control		T2D Control		T2D Cγ 28 + Lupanine 20		T2D Metformin 300 + Glibenclamide 10	
	Pre Treatment	Post Treatment	Pre Treatment	Post Treatment	Pre Treatment	Post Treatment	Pre Treatment	Post Treatment
Glucose (mg/dL)	112.6 ± 5.1	114.6 ± 7.4	479.2 ± 14.0	482.2 ± 26.2	564.6 ± 29.4	352.4 ± 54.7 **	299.0 ± 50.9	227.0 ± 19.2
Triglycerides (mg/dL)	50.2 ± 5.7	50.4 ± 2.9	86.0 ± 9.1	55.8 ± 12.4	283.4 ± 35.5	75.6 ± 13.5 ***	104.2 ± 29.4	53.8 ± 12.5
T. Cholesterol (mg/dL)	50.8 ± 5.0	56.4 ± 2.8	142.0 ± 7.9	161.0 ± 16.2	276.8 ± 76.2	189.6 ± 39.6	166.6 ± 14.9	110.6 ± 6.4 **
HDL-c (mg/dL)	36.6 ± 5.0	42.6 ± 1.9	49.8 ± 11.0	56.0 ± 13.9	23.8 ± 2.0	33.2 ± 4.2	26.2 ± 2.2	30.0 ± 1.2
LDL-c (mg/dL)	7.5 ± 3.0	7.8 ± 2.8	104.6 ± 3.6	131.8 ± 9.3	238.8 ± 68.0	162.0 ± 31.5	121.4 ± 3.2	79.2 ± 3.3 *
AST (U/L)	57.6 ± 4.9	52.0 ± 2.8	248.6 ± 77.6	367.8 ± 76.0	144.2 ± 19.1	294.4 ± 31.9 *	155.0 ± 4.2	192.2 ± 20.6
ALT (U/L)	33.6 ± 3.2	28.8 ± 2.2	249.4 ± 103.2	430.4 ± 111.5	104.0 ± 10.3	231.8 ± 71.6	61.2 ± 4.8	121.2 ± 26.6
Urea (mg/dL)	37.6 ± 5.3	36.6 ± 0.9	57.2 ± 5.9	59.4 ± 4.9	61.6 ± 3.1	57.0 ± 4.2	37.0 ± 2.9	49.2 ± 4.2 *
Creatinine (mg/dL)	0.7 ± 0.0	0.6 ± 0.0	0.8 ± 0.2	0.6 ± 0.0 **	1.3 ± 0.3	0.7 ± 0.1 **	0.7 ± 0.0	0.7 ± 0.0

Values represent the mean ± SEM, *n* = 10. T. cholesterol, total cholesterol, HDL-c, high-density lipoprotein cholesterol; LDL-c, low-density lipoprotein cholesterol; AST, aspartate aminotransferase; ALT, alanine aminotransferase. * Student *t*-test: Statistically significant changes pre vs. post induction * *p* < 0.05, ** *p* < 0.01, *** *p* < 0.001.

**Table 2:** Top 10 differentially regulated coding genes.

T2D Control and T2D Treated with Cγ + Lupanine			
Upregulated		Downregulated	
Gene	Fold Change	Gene	Fold Change
*Chac1*	6.43	*Irg1*	−3.20
*Coq10b*	6.14	*Inmt*	−2.97
*Cyp2b1*	5.28	*Ly6c*	−2.97
*Insig1*	4.33	*Ly6c*	−2.87
*Cyp2b2*	3.75	*Ly6c*	−2.84
*Tsku*	3.39	*Snai2*	−2.60
*PVR*	3.32	*Rtp3*	−2.18
*Slc38a2*	3.25	*Lcn12*	−2.15
*Btg2*	3.15	*Evi2b*	−2.14
*Slc20a1*	3.09	*Nr1i3*	−2.12

T2D: type 2 diabetes, Cγ: gamma conglutin.

**Table 3:** Genes restored in T2D rats to the expression levels found in healthy conditions (gene expression levels from healthy untreated animals) after the 28 mg/kg BW Cγ + 20 mg/kg BW lupanine treatment.

Restored Genes		Restored > 50%		
*Alpl*	*Ifrd1*	*Alas1*	*Gstk1*	*Rgs4*
*Btg2*	*Jun*	*Angptl4*	*Gzmbl3*	*Rictor*
*Chka*	*Kcnk5*	*Bcl2l11*	*Hao1*	*Serpine1*
*Coq10b*	*Lcn12*	*Bmf*	*Igfbp1*	*Slc19a2*
*Cyp2b1*	*Map2k6*	*C8g*	*Inmt*	*Slc1a4*
*Cyp2b2*	*Nrip1*	*Casp12*	*Irs2*	*Slc20a1*
*Ddit3*	*Pdha1*	*Casp2*	*Kcnj11*	*Slc37a4*
*Dld*	*Pdk4*	*Ces2c*	*Kcnj14*	*Slc38a2*
*Dusp8*	*Rtp3*	*Esr1*	*Klf6*	*Tat*
*F12*	*Slc25a25*	*Fgf21*	*Myc*	*Thap1*
*Gadd45b*	*Slc3a2*	*Fgf23*	*Pcyt1a*	*Thap2*
*Gcgr*	*Snai2*	*Foxo1*	*Pdha1l1*	*Thap3*
*Gdf15*	*Tsku*	*Foxo3*	*Ppargc1a*	*Tp53inp1*
*Gem*		*G6pc*	*Rell1*	*Tp53inp2*
		*Gpcpd1*	*Rgs1*	*Zfp354a*

Restored genes indicate genes that completely (100%) reestablished their level to a healthy condition; Restored > 50% indicates genes whose restoration was near to 100% or at least 50% of reestablishment.

## Data Availability

The data discussed in this publication were deposited on NCBI’s Gene Expression Omnibus (GSE216668).

## Supplementary Materials

The following supporting information can be downloaded at https://www.mdpi.com/article/10.3390/ph16010117/s1: Figure S1: Detection of lupanine in serum and urine of Wistar rat by thin-layer chromatography; Figure S2: Gas chromatography/mass spectrometry (GC–MS) analysis of lupanine obtained from *L. albus*; Figure S3: Characterization of Cγ obtained of *L. albus* through SDS-PAGE; Figure S4: A representative evaluation of RNA quality and integrity; Figure S5: Sample signals of restored genes in transcriptional analysis console (TAC) of Affymetrix software; Table S1: Area under the curve (AUC) of Oral glucose tolerance test (OGTT) performed employing different dose combinations of the lupin compounds.
