# Supplementary material for: Combined Gamma Conglutin and Lupanine Treatment Exhibits In Vivo an Enhanced Antidiabetic Effect by Modulating the Liver Gene Expression Profile
Source: Pharmaceuticals (Basel). 2023 Jan 13;16(1):117. doi: 10.3390/ph16010117 (PMC9867068; doi:10.3390/ph16010117)
Supplement: Supplementary file 1 [file pharmaceuticals-16-00117-s001.zip › pharmaceuticals-2095458-supplementary.pdf]

Combined gamma conglutin and lupanine treatment exhibits *in vivo* an enhanced antidiabetic effect by modulating the liver gene expression profile

Characterization and detection of lupanine from *Lupinus albus* by Thin Layer Chromatography (TLC).

Lupanine perchlorate crystals were resuspended in 1 ml of distilled water, 4 drops of 1 M ammonium hydroxide, and 1 ml of dichloromethane were added. The mixture was stirred and allowed to rest for a few minutes until two immiscible phases were distinguished, the lower phase (dichloromethane) was separated with a Pasteur pipette. Confirmation of lupanine isolation in the form of perchlorate was performed by Thin Layer Chromatography (TLC). For the TLC, aluminum plates covered with silica gel 60 GF254 (Merck) were used, as a mixture of shift dichloromethane:methanol:NH_4OH in a proportion of 8:2:0.5 and as a revealing agent, the Dragendorff reagent was used. The presence of the quinolizidine alkaloid lupanine was evaluated in serum and urine from rats treated with lupanine in TLC, Figure S1.

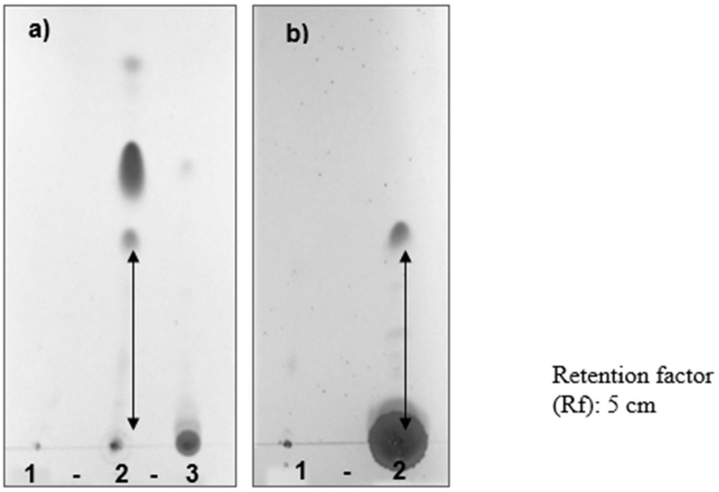

Figure S1. Detection of lupanine in serum and urine samples from Wistar rats by TLC. In panel a, lane 1 represents a serum sample after one hour of the administration of 20 mg/kg BW of lupanine; lane 2, corresponds to the detection of lupanine obtained after its extraction; and lane 3 corresponds to an urine sample after three hours of the oral lupanine administration. In panel b, a TLC replica with more sample volume in either serum (1) or urine (2) after the oral lupanine administration is shown.

In Figure S2, the upper panel shows the purity of lupanine, which yielded 98%. Below, in the same Figure, we include a representative GC-MS spectra of the lupanine obtained from *L. albus* according to the methodology described by Wink et al. (1995). Lupanine was isolated from *L. albus* species that contains mainly (+)-lupanine. Information about purity and the patent on the isolation procedure is also included (WO 1995032968 A1 also published as DE 4418618 C1, EPO 760817 A1, inventors Rainer Oeh, Klaus Rieblinger, Michael Wink, 1995).

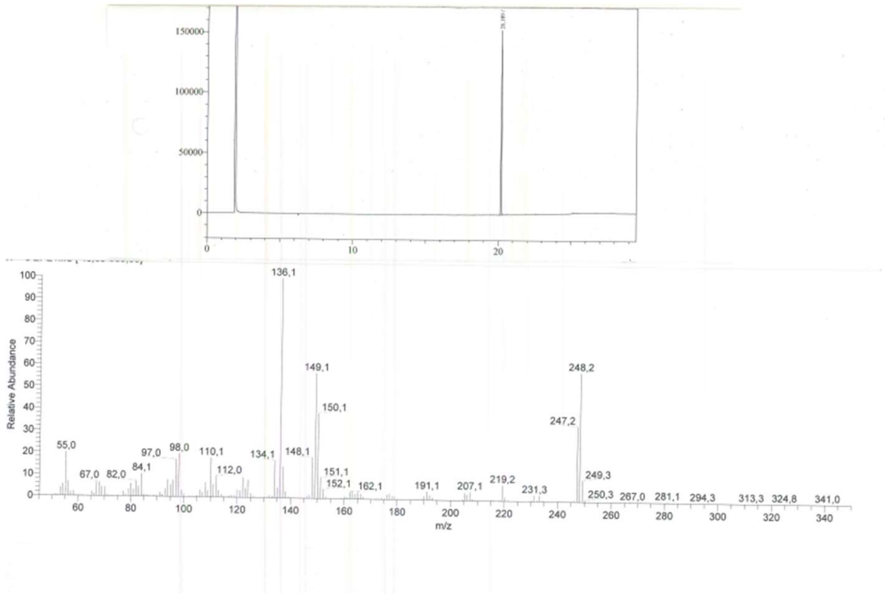

Figure S2. Gas chromatography – mass spectrometry (GC-MS) analysis of lupanine obtained from *L. albus.*

Isolation and characterization of Cγ from *Lupinus albus*

The Cγ isolated protein fraction was characterized by Sodium Dodecyl Sulfate polyacrylamide gel electrophoresis (SDS-PAGE). Under non-reducing conditions, a defined and predominant band of ~ 49 kDa that corresponds to the monomer of Cγ was observed, while in reducing conditions (in the presence of β-mercaptoethanol) two major bands of ~30 and ~17 kDa were found, corresponding to the subunits of the monomer, Figure S3.

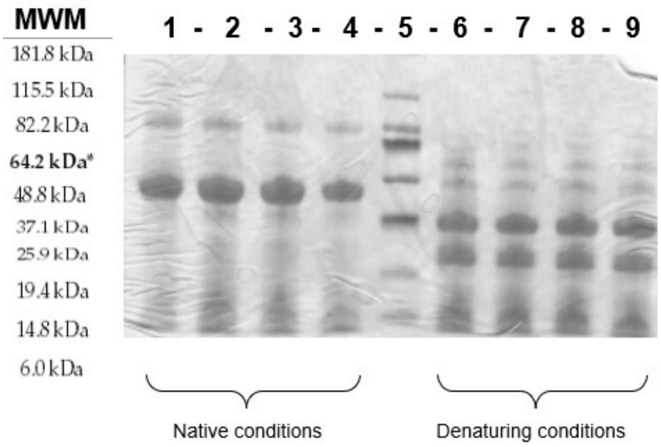

Figure S3. Characterization of Cγ obtained of *L. albus* through SDS-PAGE. Samples of Cγ (2 mg) in 200 µl of Laemmli loading buffer were resuspended. In the first four lanes, the bands corresponding to the Cγ monomer are observed under non-reduction conditions. In the central lane (5) the molecular weight marker (MWM) (benchmark prestained protein ladder) was loaded, and to the right of it, there are four samples of Cγ under reducing conditions (two bands corresponding to the two protein subunits).

Table S1. Area under the curve (AUC) of *Oral glucose tolerance test (OGTT)* performed employing different dose combinations of the lupin compounds.

Individual dose treatment (Tx)<br>Healthy rats	AUC untreated animals (Ctr)	Delta AUC untreated animals (Ctr)	AUC with treatment animals (Tx)	Delta AUC with treatment animals (Tx)	Percentage of AUC decrease (Tx *vs* Ctr)
**Cγ 28 Healthy**	14199.00	5823.00	14061.00	3093.00	46.90
**Cγ 120 Healthy**	14199.00	5823.00	14214.00	2166.00	62.80
**Lupanine 10 Healthy**	14136.00	4152.00	15813.00	5037.00	(-) 21.3
**Lupanine 20 Healthy**	13792.50	5302.50	13890.00	4800.00	9.50
**Lupanine 30 Healthy**	14136.00	4152.00	15795.00	4635.00	(-) 11.6
**Individual dose Tx in diabetic rats**					
**Cγ 28 diabetic**	50310.00	-1890.00	52867.50	-9652.50	80.40
**Cγ 120 diabetic**	48750.00	-1842.00	50067.00	-13773.00	86.60
**Lupanine 10 diabetic**	51791.25	371.25	46451.25	-9138.75	91.06
**Lupanine 20 diabetic**	52485.00	-2475.00	53636.25	-4803.75	48.50
**Lupanine 30 diabetic**	51791.25	371.25	54731.25	-4818.75	46.19
**Combined Tx in healthy rats**					
**Cγ 28 + lupanine 10**	14585.00	6065.00	13925.00	4165.00	31.30
**Cγ 28 + lupanine 20**	14827.50	4897.50	13323.75	1833.75	**62.60**
**Cγ 28 + lupanine 30**	14242.50	5662.50	17265.00	5415.00	4.40
**Cγ 75 + lupanine 20**	14585.00	6065.00	14812.50	5362.50	11.60
**Cγ 120 + lupanine 10**	14827.50	4897.50	15157.50	4087.50	16.50
**Cγ 120 + lupanine 20**	14242.50	5662.50	14208.75	3678.75	35.00
**Cγ 120 + lupanine 30**	14827.50	4897.50	13050.00	2970.00	39.40
**Triplicates combined doses Tx assay in healthy rats**					
**Cγ 28 + lupanine 20 Exp 1**	14827.50	4897.50	13323.75	1833.75	**62.60**
**Cγ 28 + lupanine 20 Exp 2**	14199.00	5823.00	14241.00	3033.00	**47.90**
**Cγ 28 + lupanine 20 Exp 3**	14199.00	5823.00	13677.00	1773.00	**69.60**
**Cγ 120 + lupanine 20 Exp 1**	14242.50	5662.50	14208.75	3678.75	35.00
**Cγ 120 + lupanine 20 Exp 2**	14199.00	5823.00	12882.00	2490.00	57.20
**Cγ 120 + lupanine 20 Exp 3**	14199.00	5823.00	12594.00	3594.00	38.30
**Combined doses Tx in diabetic rats**					
**Cγ 28 + lupanine 20**	51791.25	371.25	47655.00	-10095.0	**96.50**
**Cγ 120 + lupanine 20**	44636.25	-3153.75	49860.00	-4740.00	33.50
**Pharmacological Tx in diabetic rats**					
**Glibenclamide 10**	51791.25	371.25	47156.25	-7803.75	77.45
**Metformin 50**	51791.25	371.25	51585.0	-6675.0	66.24
**Metformin 300**	51791.25	371.25	41310.00	-8370.00	83.07
**Glib 10 + Met 50**	51791.25	371.25	49612.5	-7867.5	78.08
**Glib 10 + Met 300**	51791.25	371.25	41895.00	-9705.00	**96.32**

Optimal RNA Integrity Number of liver RNA samples from experimental groups.

The integrity of the total RNA isolated from liver tissue from healthy control group, T2D control group, and T2D treated with Cγ + lupanine group was evaluated in a 2100 Bioanalyzer equipment (Agilent, USA). The minimum RNA Integrity Number (RIN) considered to be processed using the Clariom D rat microarray chips was >6.5; the samples employed for these experiments yielded a RIN in the range of 8.80 to 9.90. RNA quality performed in a digital gel electrophoresis is also shown in the Figure S3.

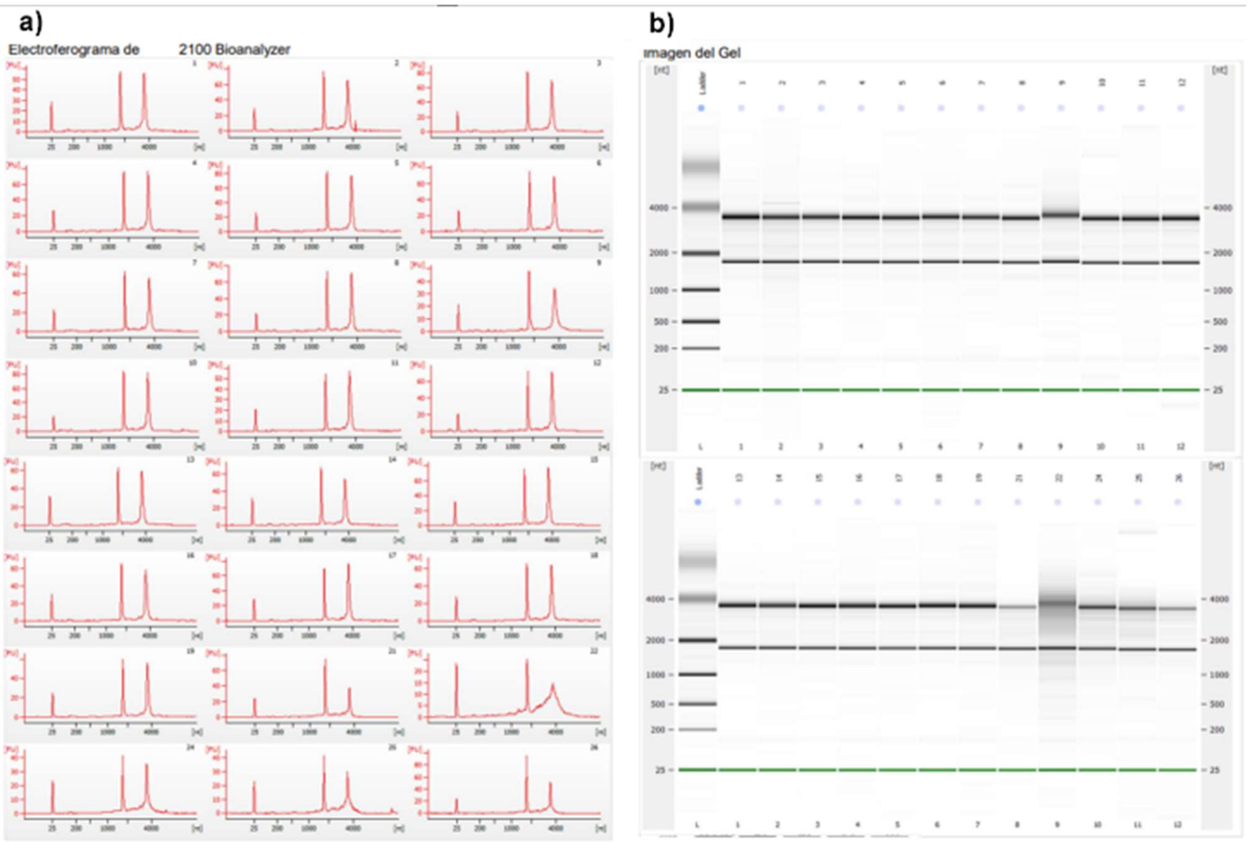

Figure S4. Representative evaluations of RNA quality and integrity of samples from the healthy group, T2D untreated group, and T2D lupin-treated group are shown. In panel a), we show data obtained from the electropherograms and in panel b) we include the results from the digital gel electrophoresis.

Gene expression levels of genes, which restored to a healthy condition level

In T2D animals, we identified that some genes were restored to the same expression levels found in a healthy condition (gene expression levels from healthy untreated animals) after the 28 mg/kg BW Cγ + 20 mg/kg BW lupanine treatment. We compared the group sample signals (expression level) from each gene using the transcriptome analysis console (TAC).

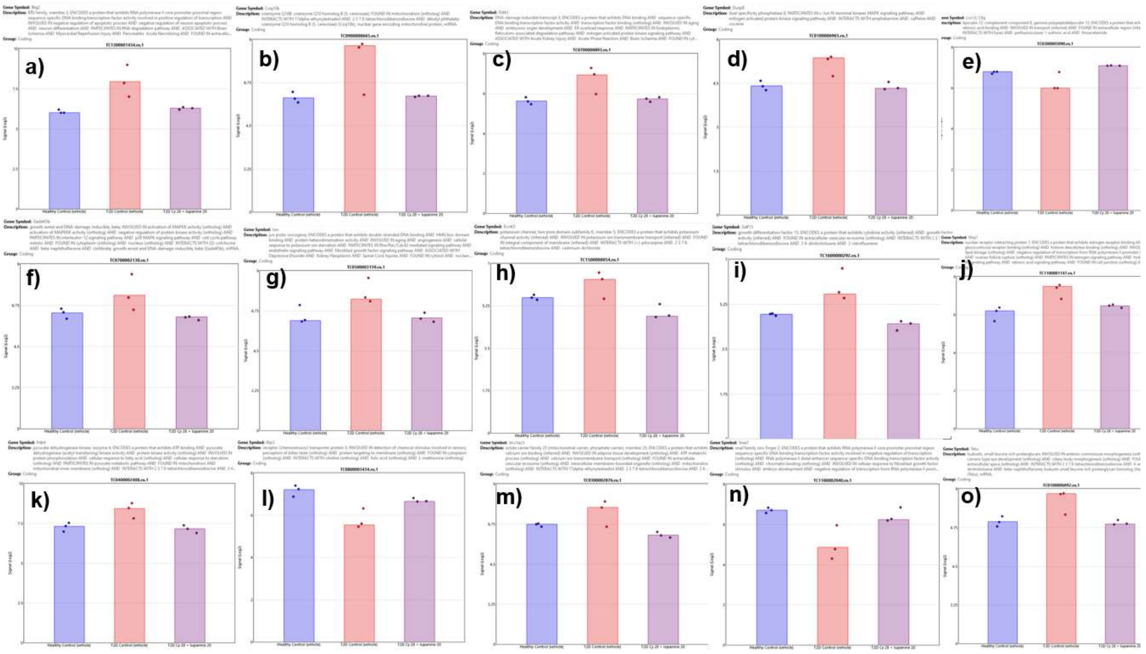

Figure S5. Sample signals of restored genes in Transcriptional Analysis Console (TAC) of Affymetrix software. Bars show Healthy untreated group (blue), T2D untreated group (pink) and, T2D group treated with Cγ 28 + lupanine 20 mg/kg (purple bars), **a)** *Btg2* **b)** *Coq10b* **c)** *Ddit3* **d)** *Dusp8* **e)** *Lcn12* **f)** *Gadd45b* **g)** *Jun* **h)** *Kcnk5* **i)** *Gdf15* **j)** *Rtp3* **k)** *Pdk4* **l)** *Rtp3* **m)** *Slc25a25* **n)** *Snai2* **o)** *Tsku.*
